# Local and global effects of sedation in resting-state fMRI: a randomized, placebo-controlled comparison between etifoxine and alprazolam

**DOI:** 10.1038/s41386-024-01884-5

**Published:** 2024-05-31

**Authors:** Simon Wein, Marco Riebel, Philipp Seidel, Lisa-Marie Brunner, Viola Wagner, Caroline Nothdurfter, Rainer Rupprecht, Jens V. Schwarzbach

**Affiliations:** Department of Psychiatry and Psychotherapy, University of Regensburg, Universitätsstrasse 84, Regensburg, 93053 Germany

**Keywords:** Neuroscience, Medical research

## Abstract

TSPO ligands are promising alternatives to benzodiazepines in the treatment of anxiety, as they display less pronounced side effects such as sedation, cognitive impairment, tolerance development and abuse potential. In a randomized double-blind repeated-measures study we compare a benzodiazepine (alprazolam) to a TSPO ligand (etifoxine) by assessing side effects and acquiring resting-state fMRI data from 34 healthy participants after 5 days of taking alprazolam, etifoxine or a placebo. To study the effects of the pharmacological interventions in fMRI in detail and across different scales, we combine in our study complementary analysis strategies related to whole-brain functional network connectivity, local connectivity analysis expressed in regional homogeneity, fluctuations in low-frequency BOLD amplitudes and coherency of independent resting-state networks. Participants reported considerable adverse effects such as fatigue, sleepiness and concentration impairments, related to the administration of alprazolam compared to placebo. In resting-state fMRI we found a significant decrease in functional connection density, network efficiency and a decrease in the networks rich-club coefficient related to alprazolam. While observing a general decrease in regional homogeneity in high-level brain networks in the alprazolam condition, we simultaneously could detect an increase in regional homogeneity and resting-state network coherence in low-level sensory regions. Further we found a general increase in the low-frequency compartment of the BOLD signal. In the etifoxine condition, participants did not report any significant side effects compared to the placebo, and we did not observe any corresponding modulations in our fMRI metrics. Our results are consistent with the idea that sedation globally disconnects low-level functional networks, but simultaneously increases their within-connectivity. Further, our results point towards the potential of TSPO ligands in the treatment of anxiety and depression.

## Introduction

Benzodiazepines are highly effective in treating stress-related conditions due to their potent and fast-working anxiolytic properties via positive allosteric modulations on *α* and *γ* binding sites of the GABA_A_ receptor [1, 2]. However, benzodiazepines exhibit short and long-term side effects, such as sedation, cognitive impairment as well as tolerance and abuse potential [3–5]. Consequently, pharmacological alternatives are needed for clinical practice. Etifoxine targets *α*2-containing GABA_A_ receptors subunits, binds to the translocator protein (18 kDa) (TSPO) and putatively acts via the formation of endogenous neurosteroids [6]. Thereby it may constitute such an alternative as it creates fewer and less pronounced side effects while being comparatively effective in terms of reducing anxiety [7–10]. Neurosteroids, if applied exogenously, e.g. in form of brexanolone and zuranolone, have recently shown promise in quickly alleviating postpartum and major depressive disorder symptoms [11, 12]. The clinical anxiolytic efficacy and non-inferiority compared to benzodiazepines has been addressed in several studies where etifoxine showed fewer disruptive side effects like sedation and cognitive impairment [7, 8, 13]. The impact of the drug on neural networks and how this may yield such a seemingly more favorable side effect spectrum is yet unknown [2, 14].

Resting-state fMRI (rs-fMRI) can track changes in brain networks related to pharmacologically induced modulations [15, 16]. Administering chloral hydrate, dexmedetomidine and propofol revealed decreases in connection density and efficiency in whole-brain networks during anaesthesia in rs-fMRI [17–19]. In a study of localized functional connectivity in rs-fMRI administering alprazolam 1.5 hrs before measurement increased local activity coherence in sensory- and sensory-integration areas [20]. Administering diazepam for 7 and 8 days (last dose 1 h before measurement) [21] or midazolam intravenous during measurement [22] yielded an increase of within-network connectivity mainly in low-level sensory networks. Additionally, midazolam in adults and thiopental in children elevated low-frequency amplitudes (<0.05 Hz) in the visual, sensorimotor and auditory resting-state network during intravenous application [23, 24]. Within the default network reduced functional connectivity in the posterior cingulate cortex was reported during sedation [25]. A review of midazolam effects summarized that altered consciousness with midazolam is, in general, accompanied by decreased cerebral blood flow in the thalami and in precuneus regions [15]. Moreover, it is hypothesized that sedation with midazolam mainly alters functional connectivity in higher-order brain areas while areas concerned with primary sensory functions tend to remain unaffected, or even display elevated within-network connectivity [22].

In our study we used rs-fMRI and behavioural assessments to compare neural correlates and side effects elicited by a TSPO ligand (etifoxine) and a benzodiazepine (alprazolam) in a three-armed, double-blind, placebo-controlled repeated-measures study. We compared modulatory effects of alprazolam, etifoxine, and a placebo with respect to a baseline measurement at the beginning of the study. We employed optimized multi-band fMRI sequences [26] and combined several different rs-fMRI analysis strategies to study their effects at high spatiotemporal resolution and across different scales. We investigated alterations of the functional network properties on a whole-brain level and subsequently explored how treatments differentially modulate connectivity individual brain regions [27]. We then studied changes in local connectivity by means of regional homogeneity (ReHo) [28] and examined localized modulations in fractional amplitude of low-frequency fluctuations (fALFF) of the BOLD signal [29]. Finally, we incorporated independent component analysis (ICA), a multivariate and data-driven technique to extract individualized resting-state networks [30], to study changes of within-network BOLD activity synchronization.

## Materials and methods

### Study participants

34 healthy male participants between the age of 18 and 55 years participated in this study (Table S1). Because it was the first neuroimaging study including etifoxine, we were not able to estimate an effect size in advance and the number of participants was selected based on practical constraints including study duration and throughput of participants. All participants were screened by a physician for the absence of physical and psychiatric disorders by physical examination and the German version of the Mini-International Neuropsychiatric Interview (MINI) [31].

### Study design

We performed a double-blind, placebo-controlled study using a repeated-measures design with fully balanced order of treatments to mitigate potential carry-over effects (trial registration: https://drks.de/search/de/trial/DRKS00020267). The study was approved by the local ethics committee (approval number 18-1197-111) and the National Institute for Pharmaceutical Security (BfArM, Eudra-CT-number: 2018-002181-40). Participants gave their written informed consent at the beginning of the experiment. Data was collected during the period 06.07.2020–17.12.2021. Subjects underwent four measurement sessions in total, starting with a baseline followed by a pseudo-randomly assigned counterbalanced medication intake of placebo, alprazolam (1.5 mg/d in 3 doses of 0.5 mg) or etifoxine (150 mg/d in 3 doses of 50 mg) for 5 days, each with at least 7 days washout phases between medications. Based on known kinetics of etifoxine and alprazolam (respective half-life times: 20 h for active metabolite of etifoxine and 15 h for alprazolam) more than 98% of the respective substance should be eliminated after six days [32], yielding negligible carry-over effects mitigated by counterbalancing. Subjects ingested the last dose at 12:00 a.m. of treatment day 5 and underwent fMRI measurement 1 h later (maximum plasma levels of etifoxine and alprazolam are expected after maximum 2 h). Based on pharmacokinetic assumptions relative serum levels at the start of the MRI session accumulate within 5 days to ~342 % (equivalent to 171 mg oral dose) for etifoxine and ~274 % (equivalent to 1.37 mg oral dose) for alprazolam. Order of treatment (placebo, alprazolam, etifoxine) was fully counterbalanced, yielding groups of six participants for each of the six possible orders. The random allocation sequence was generated using SAS 9.4 and the procedure proc plan by the Center for Clinical Trials of the University Hospital Regensburg. Since we only included 34 participants, two sequences were only realized five out of six times, as shown in the CONSORT flow diagram in Fig. S1.

All members of the study personnel enrolled participants, and all participants were assigned to all medications in this repeated measurement design. During the period of the study, neither participants nor study personal knew about the assignment of medication, which was delivered in numbered containers. The study participants were instructed to refrain from consuming alcohol, operating a car or heavy machinery, from smoking more than 5 cigarettes per day, and from consuming caffeine prior to the fMRI session.

### Self-reported side effects

Each day of medication, 1.5 h after intake, participants were required to report side effects. These side effects included dizziness, skin reactions, changes in appetite (either decreased or increased), confusion, hallucinations, tantrums, sleeplessness, nervousness, headaches, constipation, nausea, vertigo, inner restlessness, sleepiness, concentration problems, alterations in libido, and fatigue. Participants used a rating scale from 0 (no side effect) to 3 (strong side effect) to indicate the severity of these symptoms.

### MRI acquisition

Functional MRI data were collected with a Siemens Magnetom Prisma 3T Scanner at the University of Regensburg. Participants were instructed to relax and stay awake during the resting-state fMRI (rs-fMRI) session, while keeping their eyes closed. Based on pre-studies on optimizing the signal to noise ratio of echoplanar imaging (EPI) protocols [26], we used an EPI multi-band sequence (multi-band factor 4) with a repetition time (TR) of 1000 ms, echo time (TE) of 30 ms and a flip angle (FA) of 6° for acquiring functional images. During a scanning time of 22 min in total 1320 volumes were collected, with a field of view (FoV) of 192 mm × 192 mm, an acquisition matrix (AM) of 64 × 64, and an isotropic voxel size of 3 mm. Field map images were collected by using a double-echo spoiled gradient echo sequence, with TR = 715 ms, TE = 5.81/8.27 ms, FA = 40°, with an isotropic voxel size of 3 mm, generating a magnitude image and two-phase images. High-resolution T1-weighted images were acquired using a Magnetization Prepared Rapid Gradient Echo (MP-RAGE) sequence, with a TR = 1919 ms, TE = 3.67 ms, FA = 9°, AM = 256 × 256 and FoV = 250 mm × 250 mm.

### MRI preprocessing

For processing the functional and structural imaging data, we used the processing pipeline provided by fMRIPrep^1^ (version 20.2.4) [33] which included bias field correction, motion correction based on MCFLIRT [34], slice timing correction and projection onto the cortical surface, generating high-resolution grayordinate time courses in the symmetric fsLR standard space [35]. A summary of the detailed fMRIPrep preprocessing steps is provided in supplement II. Prior to the ReHo, fALFF and ICA analysis we smoothed the fMRI data using Gaussian surface smoothing with a FWHM of 3 mm [35].

### Brain connectivity analysis

**Fig. 1:**
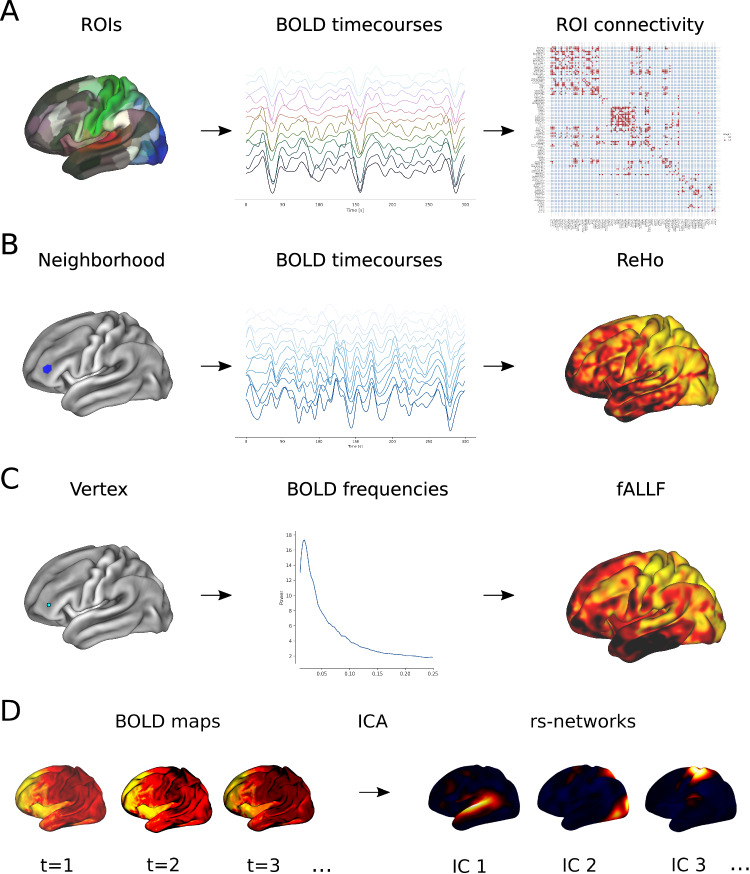
Analysis strategies used in our study to characterize activity dynamics in rs-fMRI. **A** illustrates how resting-state FC between different ROIs was computed. We used an atlas to subdivide the cortex into segregated ROIs and correlated the average BOLD time courses within each ROI, yielding a functional connectivity matrix. Using different graph theoretical measures allowed us to analyze changes of the region-wise or whole-brain connectivity. **B** shows how ReHo maps were derived from rs-fMRI data. For each vertex on the surface the neighbouring vertices within a pre-defined radius were selected to define a local neighbourhood (marked in blue in this example). By computing the similarity between all time courses within this neighbourhood, we obtained a ReHo value for each vertex on the surface. **C** illustrates the steps to compute a fALFF map from rs-fMRI data. For each vertex, the power spectrum of its BOLD time course was computed, and fALLF resulted from the ratio of the low-frequency power to the power of the entire frequency range. **D** shows the basic principle of ICA of rs-fMRI data. Spatial-temporal activity maps acquired during a session at different timepoints (t) were decomposed by ICA into a set of spatially independent components (ICs), representing coherently activated resting-state networks.

We first studied differences in resting-state functional connectivity (FC) on the whole-brain level between different pharmacological treatments. We subdivided each hemisphere into 180 regions of interest (ROIs) defined by Glasser et al. [36] and computed the average activity time course within each region and filtered the resting-state BOLD signal within the 0.01 Hz - 0.1 Hz low-frequency range. FC strength was computed with the Pearson correlation coefficient between time courses of pairs of regions to obtain a 360 × 360 FC matrix for each rs-fMRI session that was thresholded at different levels *σ* to obtained a binarized connectivity matrix (*σ* = [0.5, 0.6, 0.7]), yielding a range of reasonable and discriminative functional connectivity densities (on average 55.9%, 39.2%, and 22.0% of possible connections, respectively). This workflow is illustrated in Fig. 1A. Our goal was to study differences in functional brain networks by analyzing their graph theoretical properties such as edge density, as well as global and local network efficiency [27]. Additionally we studied the interconnectedness of high-degree nodes by analyzing the rich-club coefficient of the network [37]. While these graph measures reflect changes of the functional network on the whole-brain level, we additionally investigated in more detail alterations in connectivity of individual areas. For this purpose we also computed edge density, average connection efficiency and betweeness centrality of each brain region [27]. A detailed definition of these network metrics is additionally provided in Supplement I.

### Regional homogeneity

While classical FC analysis offers us a possibility to study long-distance relationships between segregated brain areas, regional homogeneity (ReHo) can supplement such analysis by quantifying local connectivity across the cortex at a scale of millimetres [28]. In fMRI, ReHo is defined as the temporal coherence or synchrony of the BOLD signal of neighbouring voxels or vertices. We implemented a surface-based approach, selecting for each vertex its k-hop neighbouring vertices in the fsLR standard space [35], and quantified ReHo by computing the average Pearson correlation between these neighbouring BOLD time courses (Fig. 1B). This metric allowed us to study the changes in coherence of spontaneous resting-state activity between different treatments.

### Low-frequency fluctuations

Classical connectivity-based analysis does not directly provide us with information on amplitudes of brain activity in fMRI. To account for this shortcoming we additionally investigated alterations in the fractional amplitude of low-frequency fluctuations (fALFF) [29, 38]. This metric is based on the ratio of the BOLD signal’s power spectrum of low frequencies to the signal’s entire frequency range. We focused on the very-low-frequency range 0.01–0.05 Hz, which has been reported to increase during midazolam sedation and anaesthesia [23, 24, 30]. For each vertex of the surface in fsLR standard space [35] we computed the corresponding frequency spectrum (Fig. 1C) and analyzed the localized differences of related fALFF values between different medication groups.

### Independent component analysis

Independent component analysis (ICA) is a multivariate data-driven technique [23, 39] that aims to explain spatial-temporal fMRI data by a set of coherently fluctuating source components of BOLD activity, representing spatially independent brain networks (Fig. 1D). Due to inherent ambiguities in the canonical ICA model, it is not straightforward to apply this technique for group studies [40–42]. Constrained ICA (cICA) offers a robust and well-defined approach for extracting independent resting-state networks on the subject-level, which are consistent across a group of subjects and therefore allow for group inferences [43]. Besides maximizing non-Gaussianity of estimated source networks, cICA simultaneously optimizes the similarity to a reference component to obtain well-defined and consistent components across subjects. We used the 9 cortical high-resolution resting-state networks^2^ [44] as reference components for cICA. This yielded 9 resting-state networks per subject and per intervention, allowing us to compare these spatial patterns between different medications. Prior to cICA we applied a low-pass filter with a cutoff frequency at 0.2 Hz to the BOLD timecourses. A detailed description of the cICA model and optimization procedure is provided is provided in Supplement I.

## Results

### Self-reported side effects

**Fig. 2:**
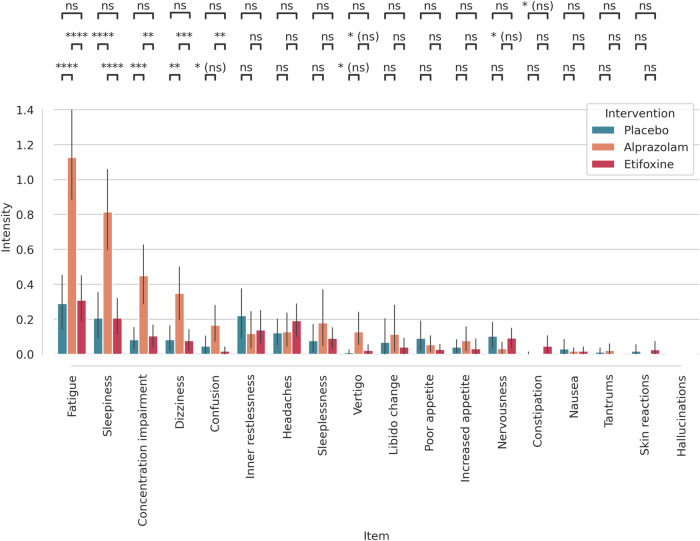
Results of the side effects questionnaire for the different interventions. Alprazolam produced stronger side effects such as fatigue, sleepiness, concentration impairment, dizziness, and confusion compared to etifoxine and placebo. Error bars represent 95% confidence intervals across subjects. Significant differences between treatments: *p ≤ 0.05, **p ≤ 0.01, ***p ≤ 0.001, ****p ≤ 0.0001, ns not significant, (ns) not significant after false discovery rate correction.

Figure 2 shows intensity scores and their respective confidence intervals for side effect-related symptoms grouped by medication and intervention. There were considerable side effects such as fatigue, sleepiness, concentration impairment, dizziness and confusion in particular related to the administration of alprazolam. For statistical testing we computed a paired t-test and applied false discovery rate (FDR) correction with *q* ≤ 0.05 to account for multiple comparisons [45]. Differential effects between alprazolam, etifoxine, and placebo are indicated by pairwise comparisons at the top of Fig. 2. Respective *p* values and effect sizes are listed in Supplement I table S2.

### MRI results

**Fig. 3:**
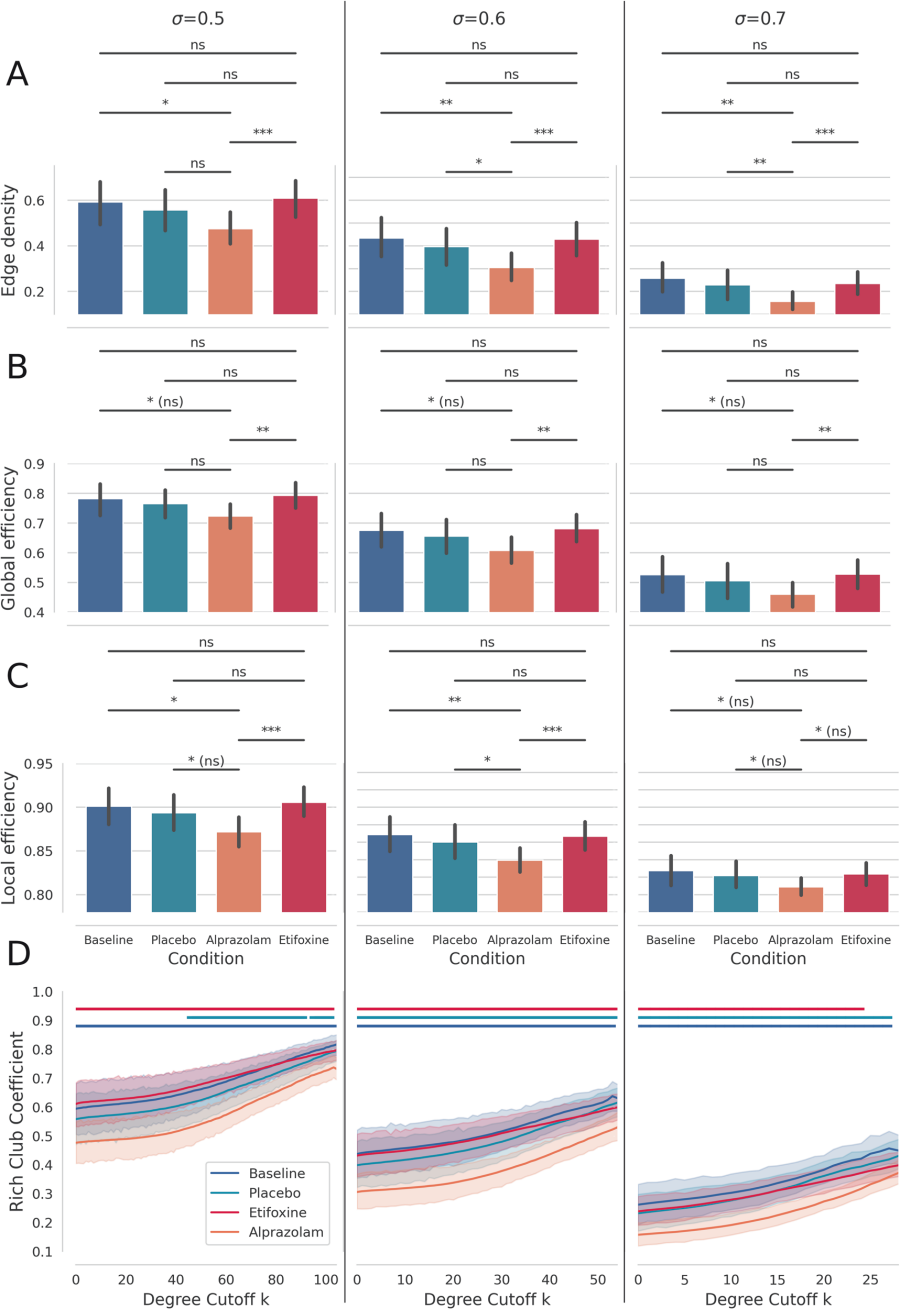
**A**–**C** Average edge density, global efficiency and local efficiency of the FC network under different medications andl for different correlation thresholds *σ*. We found reduced edge density and reduced global and local efficiency in the alprazolam condition compared to baseline, and the placebo and etifoxine conditions. In (**D**) a reduction of the rich-club coefficient due to administration of alprazolam can be observed. Regions, where the difference to alprazolam becomes significant, are indicated in bars above, with the respective colours for the placebo, etifoxine and baseline condition. Error bars represent 95% confidence intervals across subjects. Significant differences between conditions in (**A**–**C**): *p ≤ 0.05, **p ≤ 0.01, ***p ≤ 0.001, ns not significant, (ns): not significant after false discovery rate correction. Bars in (**D**) indicate regions where a significant difference to alprazolam can be observed with *p* ≤ 0.05.

In our first analysis we studied differences of the functional brain network at a global level by comparing whole-brain edge densities of FC networks between baseline and the three different interventions. Figure 3A–C illustrates edge density, local efficiency and global efficiency of the FC network for the baseline-, placebo-, alprazolam- and etifoxine conditions respectively, for three different connectivity thresholds *σ*. For each rs-fMRI session we computed the corresponding graph measure and applied a paired t-test between fMRI sessions with different medications, including FDR correction (*q* ≤ 0.05) to control for multiple comparisons [45]. In addition, we analyzed the rich-club coefficient of the whole-brain network in Fig. 3D. For each degree cutoff *k* of the rich-club coefficient we applied a paired *t*-test and used FDR correction to account for comparing multiple groups. We analyzed the rich-club coefficient up to a maximum degree cutoff *k* where this metric could be computed for all 34 subjects. To account for testing this metric across various degree cutoffs *k*, we applied principal component analysis and tested for differences in individual loadings of this metric on the first principal component [46], since the values are highly correlated (explaining 91.9%, 93.0%, and 87.8% of the variance for the thresholds *σ* = [0.5, 0.6, 0.7] respectively).

We further preliminary explored the direct relationship between self-reported side effects and modulations in resting-state connectivity in Fig. S2. Subjects that reported stronger side effects showed a consistent but not significant tendency of reduced FC edge density, and reduced global and local efficiency.

As shown in Fig. 3A we mainly observe a significantly reduced functional edge density in the alprazolam condition, compared to the baseline, and to treatment with placebo or etifoxine. Similarly we found a tendency of reduced local and global efficiency due to alprazolam, as depicted in Fig. 3B, C, while observing no such down-modulation effects in the etifoxine condition. Figure 3D shows a significantly reduced rich-club coefficient in the alprazolam condition in comparison to the placebo, etifoxine and baseline condition for the vast majority of degree cutoffs *k* (regions with significant differences *p* ≤ 0.05 to the alprazolam condition are indicated with bars on top of the graphs). We also found an overall significant difference (*p* ≤ 0.05) in principal component loadings between alprazolam and all other conditions for all thresholds *σ*. We found no significant modulation of the rich-club coefficient in the etifoxine condition.

**Fig. 4:**
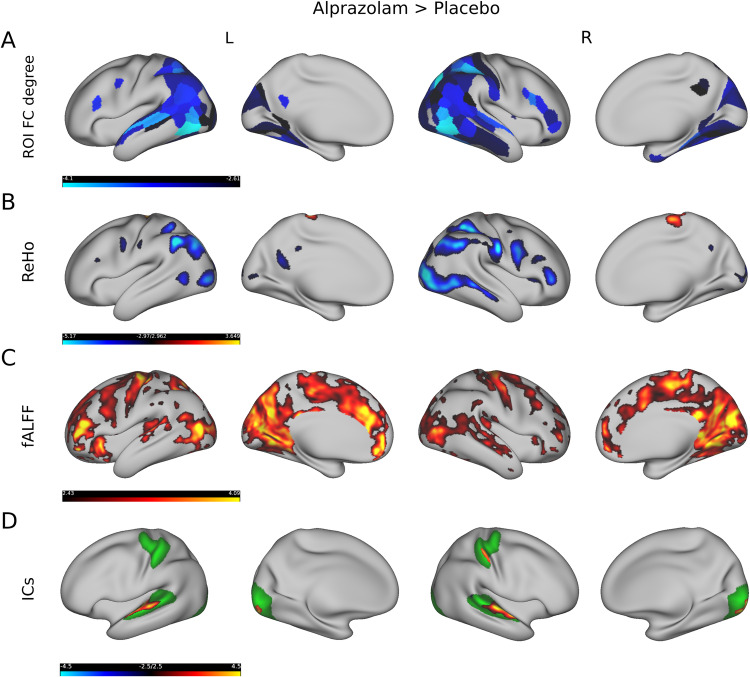
Comparison of rs-fMRI measures between alprazolam and placebo. The first row (**A**) shows the regions in the left (L) and right (R) hemisphere where FC degree was significantly lower after administering alprazolam compared to placebo. The second row (**B**) depicts several regions in which local connectivity, as defined by ReHo, was lower after administering alprazolam compared to placebo, except for one region in superior/medial aspects of somato-motor cortex. The third row (**C**) shows that low-frequency amplitudes, as derived from fALFF, were higher after administering alprazolam compared to placebo. The fourth row (**D**) illustrates alterations in ICA-based resting-state networks, which display higher connectivity after the administration of alprazolam within the temporal, occipital and right somatosensory cortex. Yellow and blue colours depict t-values (from blue, alprazolam < placebo, to yellow, alprazolam > placebo).

We then studied regional differences in functional markers between interventions and investigated differences in ROI connectivity degree, ReHo, as well as modulations in fALFF. To test for differences in those metrics between groups we employed a paired *t*-test and used FDR correction (*q* ≤ 0.05) to account for comparing multiple ROIs or vertices [45]. Figure 4A shows those regions of the multi-modal parcellation atlas [36] that significantly differed in connectivity degree after administering alprazolam compared to placebo. For this comparison we selected a moderate FC threshold of *σ* = 0.6. We observed a significant decrease in FC mainly in the superior temporal gyrus (regions A5, STSdp as described by Glasser et al. [36]), inferior parietal lobule (PGi, PGs), intraparietal sulcus (LIPd, LIPv), inferior frontal gyrus (IFSp) and middle temporal cortex (MT, MST, FST, PH). Figure 4B depicts regions that were significantly altered in their local connectivity characterized by ReHo. To compute ReHo we employed a medium-sized neighbourhood radius of 4 vertices (≈5.2 mm) in this comparison, but additionally provide results, illustrated in Fig. S4, when using a smaller neighbourhood consisting of only 2 vertices (≈2.6 mm). We found a decrease in ReHo mainly in the inferior parietal lobule (PGs, PFm), right precentral sulcus (R 6v), right inferior frontal sulcus (R IFSa), and occipital lobe (LO1, LO2, V4, V4t). Interestingly, we observed a reversed effect of increased ReHo in the precentral gyrus/primary motor cortex (4). Local differences in the low-frequency amplitudes based on fALFF are shown in Fig. 4C. For this metric we found mainly increased fALFF ranging from the middle temporal cortex (MT, MST) to the occipital lobe (V1, V2, V3) and parieto-occipital sulcus (POS1, POS2), and also in the external cingulate gyrus (p32), anterior midcingulate cortex (a32pr), inferior frontal sulcus (IFSa, a9-46v), and precentral gyrus/primary motor cortex (4). We further investigated localized differences in resting-state networks extracted by ICA. We selected all vertices within one resting-state network by thresholding the *z*-maps with ∣*z*∣ ≥ 2 and using a paired *t*-test (FDR corrected with *q* ≤ 0.05) to test for differences in these networks related to intervention. In the comparison between placebo and alprazolam we found significant differences in 3 out of 9 resting-state networks. Figure 4D shows the significantly different spatial areas of all 3 networks projected onto one cortical surface for convenience. The average resting-state networks (thresholded at ∣*z*∣ ≥ 2) across subjects are illustrated in the background in green, while locations with significantly higher/lower connectivity are highlighted in red/blue in this figure. In this comparison we observed an increase in the BOLD activity coherence in the superior temporal cortex (A6), in the right primary somatosensory cortex (R 1) and in the primary visual cortex (V1).

**Fig. 5:**
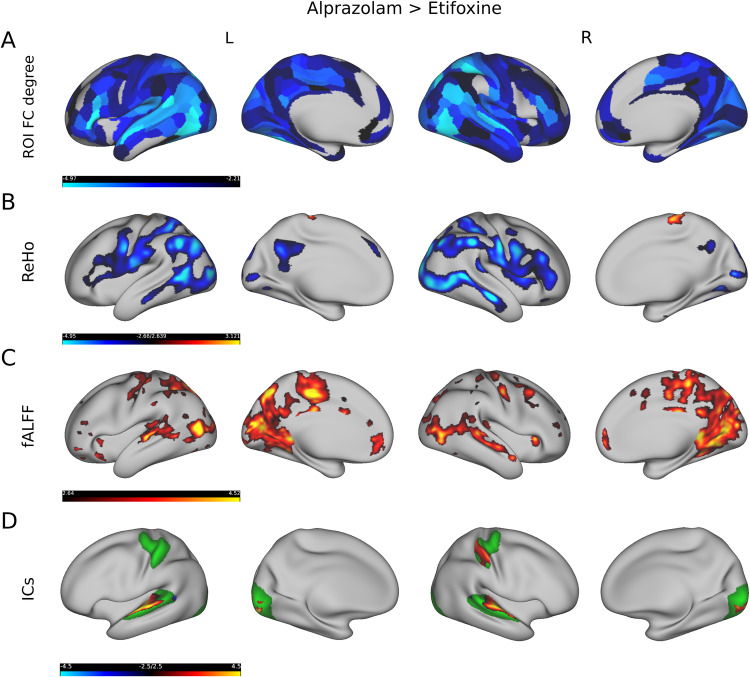
Comparison of rs-fMRI measures between alprazolam and etifoxine. The first row (**A**) shows that FC degree was significantly lower after administering alprazolam compared to etifoxine. The second row (**B**) depicts several regions in which local connectivity, as defined by ReHo, was lower after administering alprazolam compared to etifoxine. The third row (**C**) shows that low-frequency amplitudes, as derived from fALFF, were higher after administering alprazolam compared to etifoxine. The fourth row (**D**) illustrates alterations in ICA based resting-state networks, which show higher coherence in the alprazolam condition in the temporal, occipital and right somatosensory cortex. Yellow and blue colours depict t-values (from blue, alprazolam < etifoxine, to yellow, alprazolam > etifoxine).

In a comparison between alprazolam and etifoxine we found modulatory effects of connectivity degree, ReHo and fALFF spatially similar but more pronounced to the rs-fMRI metrics reported above. As shown in Fig. 5A we found lower FC for the alprazolam treatment mainly in the superior temporal gyrus (A4, A5, STV, STSdp, STSda), inferior parietal lobule (PGi, PGs), precentral and postcentral gyrus (1, 2, 3a, 3b, 4), and ranging from the occipital (V1, V2, V3) to the medial temporal lobe (MT, FST, MST, PH). Also, we noticed considerably lower FC in the inferior frontal gyrus (IFSp, 45), whereby these differences in FC strength appeared more pronounced than in the aprazolam-placebo comparison. Furthermore, as illustrated in Fig. 5B, we detected a decrease in ReHo after administering aprazolam compared to etifoxine, mainly in the inferior parietal lobule (PGs, PGi, PFop), lateral occiptal complex of the occipital lobe (PHT, FST), occipital lobe (LO1, LO2, V4, V4t), precentral sulcus (6v), inferior frontal sulcus (IFSa, IFSp, 44, 45), and posterior cingulate cortex (7m, 31pd, 31pv). We also observed a significant increase in ReHo in the precentral gyrus/primary motor cortex (4). Figure 5C shows increased low-frequency oscillations raining from the occipital lobe (V1, V2) to the middle temporal cortex (MT, MST), parieto-occipital sulcus (POS1, POS2), external cingulate gyrus (p32), precentral gyrus/primary motor cortex (4), and posterior cingulate cortex (23c, 24dd, 5mv). As illustrated in Fig. 5D, we also found differences in connectivity of 3 resting-state networks, which showed increased coherence in the superior temporal cortex (A6), right primary somatosensory cortex (R 1) and in the primary visual cortex (V1).

A comparison between alprazolam and the baseline condition is shown in Fig. S3. Here we also found a significant decrease of ROI degree for the majority of brain regions (Fig. S3A). Similar to the comparison to the placebo group, we observe mainly a decrease of ReHo (Fig. S3B), but simultaneously an increase in the precentral gyrus/primary motor cortex (4) and parieto-occipital sulcus (ProS, PreS). Further we also detect strongly increased fALFF values in the alprazolam session in comparison to the baseline condition (Fig. S3C) and found increased resting-state network coherence in low-level sensory networks (Fig. S3D). To relate these regions altered by treatment with alprazolam to commonly observed resting-state networks, we provide an overlay of all modulations with the 7 resting-state networks defined by Yeo et al. [47] in Figs. S8, S9 and S10.

In addition to analyzing differences in the connectivity degree of ROIs within the functional network, we also studied changes in the connectivity efficiency, as shown in Fig. S5. Here we observed a downmodulation of connection efficiency after administering alprazolam, wherein differences were the strongest in comparison to the etifoxine condition. As shown in Fig. S6 we found an increase in the betweenness centrality in the alprazolam condition in comparison to etifoxine in the middle (6, R FEF) and right superior frontal gyrus (R SFL, R 8BL), including the right occipital cortex (R LO1).

We observed an increase fALFF between etifoxine and baseline in left anterior cingulate and right orbitofrontal cortices (see Supplementary Fig. S7). However, we did not find such differences in our critical comparison etifoxine vs. placebo. We did not find any significant differences between the placebo and baseline conditions.

## Discussion

### Summary of findings

In this double-blind and placebo-controlled study, we used a repeated-measures design and rs-fMRI to investigate side effects and the neural correlates of a benzodiazepine (alprazolam) in comparison to a TSPO ligand (etifoxine). After administering alprazolam, we observed a considerable increase in fatigue, sleepiness, concentration problems, dizziness, and confusion, all of which are well-known side effects of benzodiazepines [3–5], which we did not find in the etifoxine condition.

To study the neural correlates of these interventions we analyzed the impact of alprazolam and etifoxine on whole-brain functional connectivity in comparison to the placebo- and baseline conditions. We observed a considerable decrease of connection density after the administration of alprazolam. Comparable decrease in whole-brain connectivity has been reported in rs-fMRI after sedation with chloral hydrate and dexmedetomidine [17, 18] and using nitrous oxide in electroencephalography [48]. The decrease of the rich-club coefficient in the alprazolam condition points out that also highly connected brain regions lose their interconnectedness. We studied in more detail regional effects of medication in terms of global connectivity. We observed decreases in connection degree and connection efficiency mainly in the inferior parietal lobule, middle frontal gyrus, postcentral gyrus and occipital lobe after administration of alprazolam in comparison to the other three conditions. Changes in the network topology under alprazolam were reflected in an increase in betweenness centrality mainly in the middle and superior frontal cortex in comparison to etifoxine. We further preliminarily explored direct relationships between FC and side effect strengths. We found that subjects that reported stronger side effects showed a consistent but not significant tendency of reduced FC edge density, and reduced global and local efficiency. To investigate this relationship in more depth, it probably would have been necessary to stronger sedate the subjects to achieve a larger range of side effects strengths, what might be interesting for future studies.

We subsequently analyzed differences in local connectivity and found a decrease in ReHo mainly in the inferior parietal lobule, precentral sulcus, inferior frontal sulcus, and occipital lobe after administration of alprazolam. Interestingly, we observed increases in ReHo in the precentral gyrus/primary motor cortex. A recent rs-fMRI study on alprazolam [20] found, similar to our results, increased local connectivity in sensorimotor cortex, but also in visual cortices. This latter difference to our findings could be due to procedural differences as participants of our study underwent rs-fMRI with their eyes closed. It is known that ReHo in the visual system is sensitive to participants having their eyes open or closed in rs-fMRI [49, 50]. We additionally found a decrease in local connectivity within frontal and parietal cortices what might be attributed to a functionally clearer definition of local neighbourhoods based on the cortex surface [28], as well as using multi-band EPI sequences, including fieldmaps, for optimizing analysis sensitivity [26].

Further, we found increased fALFF in the occipital lobe, parieto-occipital sulcus, precentral gyrus and posterior cingulate cortex related to the administration of alprazolam. Such an increase of in the very-low BOLD signal frequency range <0.05 Hz has also been observed in sedation related to anaesthesia and to the administration of midazolam in studies of Kiviniemi et al. [23, 24, 30] mainly in visual, sensorimotor and auditory resting-state networks.

Finally, we incorporated a multivariate ICA approach to assess brain network specific alterations. Similar to rs-fMRI studies on midazolam [21, 22], ICA revealed increased within-network coherence in the alprazolam condition compared to the placebo, etifoxine and baseline conditions in regions belonging to low-level sensory systems such as primary visual and superior temporal and in the primary somatosensory cortex. Our findings on alprazolam therefore align with the results of those studies and support the idea of higher coherence within sensory networks related to sedation.

These results need to be discussed in light of possible limitations. A larger number of subjects might have improved the sensitivity for etifoxine-related effects. However, the use of a repeated-measures design should mitigate decremental effects on power. In addition, the study design was optimized to detect differences in the rs-fMRI measures and self-reported side effects respectively. Analysis of potential correlations between resting-state measure changes and adverse effects beyond this level must remain exploratory. Further research building on these findings and addressing the relationship between the neural measures and side effects could include objective measures of side effects, such as concentration tests, and attempt to establish a dose-dependent relationship. Finally, for the safety of the participants, the drug dose was titrated to a moderate level, resulting in only modest behavioural effects. Therefore, conclusive evidence for behavioural correlates of the demonstrated pharmacologically induced neural effects can only be provided as a trend and thus remain hypothetical.

### Interpretation and interrelation of metrics

A recent review on midazolam [15] pointed out that lower level sensory networks show elevated activity levels under weak sedation, what is backed by the observed increase in local connectivity in sensory networks in our study. Analyzing low-frequency fluctuations we found a tendency of increased fALLF values in regions where we detected increased ReHo related to the administration of alprazolam (see Figs. S11, S12, and S13). This could indicate that more homogeneous low-frequency fluctuations due to sedation are in general accompanied by a higher local coherence of the BOLD signal. With ICA we additionally detected an increase in resting-state network coherence in the superior temporal and primary visual cortex. This higher sensitivity of ICA towards changes in within-network connectivity might be attributed to the idea of ICA of decomposing BOLD maps into isolated independent networks. However, with ICA, we did not observe any changes in higher-level networks after administrating alprazolam, while we found a considerable decrease in ReHo in such regions. In higher-level brain networks only considering within-network coherence might be insufficient to capture more complex interactions with other networks. While each rs-fMRI metric captures only one aspect of the complex spatiotemporal dynamics, analyzing multiple metrics simultaneously helps to better preserve the different characteristics of the BOLD signal. While functional whole-brain connectivity reflects global aspects of interactions between brain areas, ReHo and fALFF preserve local characteristics of the BOLD signal. Independent component analysis provides an additional data-driven method to investigate individual resting-state networks. Therefore in an exploratory fMRI study like this, where effects in fMRI are rather unknown, it is beneficial to simultaneously explore the local and global modulations of pharmacological interventions simultaneously.

The loss of global functional connectivity in low-level sensory regions under sedation could relate to several well-known behavioural side effects of alprazolam. Observed modulations in the motor and visual cortices might be connected to motor coordination impairments and visualspatial and visuomotor abilities related to benzodiazepines [51–53]. The decrease of global and local connectivity in high-level region might be connected to impairments in cognitive domains such as attention, working memory and semantic processing, which are affected by long-term treatments with benzodiazepines [54, 55].

Corresponding to the behavioural assessments in the etifoxine condition we did not find significant alterations in any rs-fMRI metric. Several clinical studies have reported an anxiolytic effect of etifoxine for a patient population, postulating a benzodiazepine comparable efficiency in psychopathological circumstances [7–10]. Therefore, for future studies in this field it might be interesting to investigate the effect of TSPO ligands in rs-fMRI for a clinical cohort of subjects with potentially different neurosteroid levels. Our study so far suggests that the favorable clinical side effect profile of etifoxine can be underpinned by neurophysiological data, as none of the typical rs-fMRI markers of sedation were affected by etifoxine.

### Pharmacological mechanisms

The pharmacodynamic properties of etifoxine differ in several aspects from benzodiazepines [56]. Benzodiazepines allosterically bind to GABA_A_ receptors containing the *α*1, *α*2, *α*3 or *α*5 subunits potentiating postsynaptic inhibition [57]. Etifoxine targets *α*2 containing GABA_A_ receptors subunits and binds to the translocator protein (TSPO) [6] and enhances neurosteroidogenesis [58, 59]. Although changes in GABAergic signalling have been demonstrated in vitro within 1 h [58, 59] of direct application of etifoxine, the treatment duration of 5 days in our study may still have limited the substance effects. The formation of neurosteroids by etifoxine could account for a different effect profile in the brain, as neurosteroids not only affect phasic inhibition via synaptical GABA_A_ receptors, but even more tonic inhibition via extrasynaptic delta subunits containing GABA_A_ receptors [57, 60]. Neurosteroids are even more prone to exert tonic inhibition and the corresponding receptors are expressed mainly within the cerebellum, thalamus and hippocampus [61]. As such, although both substances are believed to act on the GABA neurotransmitter system, their basic differences in target structures and dynamically reflect the differences in their impact on neurophysiological measures like functional connectivity. A specific network stabilization against perturbations or pathological set point alterations, e.g., by tonic postsynaptic GABAergic activity, might explain the lack of resting-state network activity effects of etifoxine in healthy participants. In fact, a concordant picture could be drawn by the use of transcranial magnetic stimulation measures examining etifoxine and alprazolam effects in our group [62].

### Conclusion

Comparing etifoxine and alrapzolam in rs-fMRI and studying their effects by combining complementary analysis strategies allowed us to understand neural correlates of sedative side effects in greater detail. The observed decrease in global connectivity and the increase in local connectivity in low-level sensory regions support the idea of a reduction in global integration and sensory network decoupling under sedation. In addition, the absence of sedation-typical behavioural and neurophysiological side effects of etifoxine supports the concept of neurosteroidogenic compounds as psychopharmacological agents for the treatment of anxiety and depression.

## Supplementary information

Supplement 1

Supplement 2

## Data Availability

The datasets during and/or analyzed during the current study are available from the corresponding author on reasonable request.

## Footnotes

1 https://fmriprep.org/en/stable/.

2 https://github.com/martahedl/Updated-atlas-for-corresponding-brain-activation-during-task-and-rest.
