# Supplementary material for: Local and global effects of sedation in resting-state fMRI: a randomized, placebo-controlled comparison between etifoxine and alprazolam
Source: Neuropsychopharmacology. 2024 May 31;49(11):1738–48. doi: 10.1038/s41386-024-01884-5 (PMC11399242; doi:10.1038/s41386-024-01884-5)
Supplement: Supplementary file 1 — Supplement 1 [file 41386_2024_1884_MOESM1_ESM.pdf]

Local and global effects of sedation in resting-state fMRI: A randomised, placebo-controlled comparison between etifoxine and alprazolam

Simon Wein[1], Marco Riebel[1], Phillip Seidel[1], Lisa-Marie Brunner[1], Viola Wagner[1], Caroline Nothdurfter[1], Rainer Rupprecht[1], and Jens V. Schwarzbach[1]

[1]Department of Psychiatry and Psychotherapy, University of Regensburg, Regensburg, Germany

Supplementary Information

Supplement I: Definitions

Graph analysis In our study we investigated modulations of the functional network related to different medications. *Functional connectivity* (FC) between two brain regions i and j was thereby computed as the Pearson correlation coefficient r_{ij} between the averaged BOLD signals of these regions. When computing these correlation values between all N regions, a FC network can then be characterized by an adjacency matrix $\mathbf{A} \in \mathbb{R}^{N \times N}$, whereby one entry a_{ij} of this matrix describes the FC strength between brain region i and j. Based on this network the *degree* d_i of a brain region i in the network can then be defined as:

$$d_i = \sum_{j=1}^{N} a_{ij} \tag{1}$$

The *edge density* ρ_A of a whole network can be described as the ratio of all possible connections to connections that are actually present:

$$\rho_A = \frac{\sum_j a_{ij}}{N(N-1)} \tag{2}$$

The *shortest path length* d_{ij} between node i and node j in a network can be defined as the minimum number of edges traversed in an optimal path between those nodes. Based on this definition, the connection *efficiency* E_{ij} between two nodes i and j can be derived [10]:

$$E_{ij} = \frac{1}{d_{ij}} \tag{3}$$

Further the *global efficiency* of a network can then be computed as the average efficiency between all pairs of nodes:

$$E_{global} = \frac{\sum_{j \neq i} E_{ij}}{N(N-1)} \tag{4}$$

Thereby global efficiency characterizes the inter-connectedness of nodes in a graph, and presents a measure of integration or ability of parallel information transfer [1]. Futher the *local efficiency* of a node i is based on the following definition:

$$E_{local,i} = \frac{\sum_{j \neq k \in G_i} E_{jk}}{d_i(d_i - 1)} \tag{5}$$

where G_i is the sub-graph of node i, and d_i its degree. The local efficiency of a network can then be computed as the average node local efficiency:

$$E_{local} = \frac{\sum_i E_{local,i}}{N} \tag{6}$$

Local efficiency characterizes the inter-connectedness of each sub-graph and represent a measure of segregation or fault tolerance, indicating how efficient the communication in a network remains in absence of a node i [1]. The centrality of a node i in a graph G can be described by *betweenness centrality*, defined as the sum of the fraction of shortest paths between two nodes j and k that pass through node i:

$$c_B(i) = \sum_{j,k \in G} \frac{\sigma(j,k|i)}{\sigma(j,k)} \tag{7}$$

where $\sigma(j,k)$ is the number shortest paths between j and k, and $\sigma(j,k|i)$ the number of shortest paths passing through node i (other than j, k). Finally the *rich-club coefficient* of a network can be used to characterize the inter-connectedness of nodes with a high degree:

$$\Phi(k) = \frac{2E_{>k}}{N_{>k}(N_{>k} - 1)} \tag{8}$$

with $E_{>k}$ representing the number of edges of nodes with a degree larger than k and $N_{>k}$ the number of nodes with a degree larger than k [9]. If for large values of k the rich-club coefficient is close to 1, it means that high-degree nodes are well interconnected.

Regional homogenity Besides studying FC between brain regions within the whole network, we investigated local connectivity, as defined by *regional homogenity* (ReHo) measures. Thereby ReHo characterizes the synchronicity of the BOLD signal within a local neighbourhood of voxels or vertices [13]. In a neighbourhood containing in total N vertices, we computed a ReHo measure as the average Pearson correlation coefficient r_{ij} between all pairs timecourses i and j:

$$ReHo = \frac{\sum_{i \neq j} r_{ij}}{N(N-1)} \tag{9}$$

Low frequency fluctuations To analyze changes in spectral characteristics of the BOLD signal, we studied alterations in *fractional amplitude of low frequency fluctuation* (fALFF) values across the cortex [14]. For each voxel or vertex i fALFF can be computed as the ratio of the power of the BOLD signal $S_i(t)$, after being filtered with a bandpass filter $h(t)$, to the power of the unfiltered signal $S_i(t)$:

$$fALFF = \sqrt{\frac{\sum_t (h(t) * S_i(t))^2}{\sum_t S_i(t)^2}} \tag{10}$$

Here $*$ denotes the convolution operation and t the temporal index. In our study we focused on the very low frequency range $0.01Hz - 0.05Hz$, which has shown to be characteristic for sedation effects observed in resting-state fMRI [6, 7, 4].

Constrained independent component analysis To make *independent component analysis* ICA suitable for applications like multi-subject fMRI studies, an extension denoted as *constrained ICA* (cICA) has been proposed by Lu and Rajapakse [8]. Like in classical ICA, the basic goal is to estimate a set of N source components $\mathbf{y} \in \mathbb{R}^N$ from the observed data $\mathbf{x} \in \mathbb{R}^K$ by estimating a demixing/weight matrix $\mathbf{W} \in \mathbb{R}^{N \times K}$:

$$\mathbf{y} = \mathbf{W}\mathbf{x} \tag{11}$$

In our study the data is represented by the time-varying BOLD signal $\mathbf{x} = (x_1(t), \ldots, x_K(t))^T$ in all K voxels/vertices observed at different timesteps t. Statistically independent components y can be reconstructed by maximizing negentropy, which can be approximated by [2]:

$$J(y) = \rho[E\{G(y)\} - E\{G(\nu)\}]^2 \tag{12}$$

where ρ denotes a positive constant, $E\{\cdot\}$ represents the expectation value and ν is a Gaussian random variable with zero mean and unit variance. Further $G(\cdot)$ can be any non-quadratic function which can be practically selected as [3]:

$$G(y) = \frac{\log \cosh(a_1 y)}{a_1} \tag{13}$$

with $1 \leq a_1 \leq 2$. Besides maximizing an approximation of negentropy $J(y)$, cICA includes the similarity to a given reference component $r_n(t)$ as constraint into the optimization [8]. This additional constraint can be formulated as $g(\mathbf{w}) = \rho - \epsilon(y, r) \leq 0$, where ρ denotes a pre-defined similarity threshold parameter, and $\epsilon(\cdot)$ a function that measures the closeness of the estimated source component y to a reference r. In our study we selected the correlation between y and r as similarity measure $\epsilon(y, r) = E[y, r]$. Based on these definitions, the augmented Lagrangian function $\mathcal{L}(\mathbf{W}, \mu)$ for estimating N source components y_n, given N references r_n can be defined as [8]:

$$\mathcal{L}(\mathbf{W}, \boldsymbol{\mu}) = \sum_{n=1}^{N} \left(J(y_n) + \frac{\max^2\{0, \mu_n + \gamma_n g_n(\mathbf{w}_n)\} - \mu_n^2}{2\gamma_n} \right) \tag{14}$$

with $\boldsymbol{\mu} = (\mu_1, \ldots, \mu_N)^T$ denoting a set of Lagrangian multipliers, and $\boldsymbol{\gamma} = (\gamma_1, \ldots, \gamma_N)^T$ representing positive learning parameters for the penalty term. The Lagrangian function can then be maximised by simply using a gradient-based learning update rule:

$$\mathbf{W}_i = \mathbf{W}_{i-1} + \eta \frac{\partial \mathcal{L}(\mathbf{W})}{\partial \mathbf{W}} \tag{15}$$

where the update step at iteration i is controlled by the learning rate η. During the optimization the weights $\mathbf{W}$ can be normalized and decorrelated to prevent them from constantly growing and avoid that different weights $\mathbf{w}_n$ estimate the same independent component [5].

As reference components we incorporated the 9 high-resolution cortical template resting-state networks defined by Tahedl et al. [11]. This allowed us to estimate 9 corresponding resting-state networks per subject and per session. Prior to ICA we reduced the 1320 BOLD activity maps collected during one fMRI session to 30 activity maps using principal component analysis (PCA). We selected a moderate similarity threshold of $\rho = 0.3$ to obtain consistent but session specific resting-state networks. During training we set the learning rate to $\eta = 0.1$ and penalty term update parameter to $\gamma = 1$[1].

[1] https://github.com/simonvino/constrained_ICA

Supplement II: Supplementary figures and tables

Table S1: The table shows demographics of study participants.

metric	BMI [kg/m^2]	age [$years$]
mean	24.3	27.2
std	3.1	6.84
min	20.0	20
max	32.13	50

	education [*participants*]
main school (9 years)	1
secondary school (10 years)	2
high school (12 years)	33

	residential environment [*participants*]
urban	28
outskirts	6
rural	2

Table S2: The table shows p-values and effect sizes (Cohen's d) from the comparison of side effects related to treatment with alprazolam (alp), etifoxine (eti) or placebo (pla). All differences that remain significant after correcting for multiple comparisons are marked in bold font.

item	p-values			effect sizes		
	pla / alp	alp / eti	pla / eti	pla / alp	alp / eti	pla / eti
Fatigue	**2.1e-06**	**3.4e-07**	0.8171	-1.2273	1.2507	-0.0419
Sleepiness	**9.3e-06**	**6.2e-06**	1.0000	-1.0413	1.1040	-0.0000
Concentration problems	**0.0004**	**0.0012**	0.5318	-0.9162	0.8545	-0.1145
Dizziness	**0.0036**	**0.0007**	0.8827	-0.6978	0.7309	0.0268
Confusion	0.0404	**0.0042**	0.3327	-0.4705	0.6334	0.2386
Inner restlessness	0.2011	0.7246	0.2921	0.2735	-0.0664	0.2297
Headaches	0.9243	0.3648	0.2537	-0.0209	-0.2078	-0.2455
Sleeplessness	0.2477	0.2486	0.7580	-0.2742	0.2462	-0.0659
Vertigo	0.0165	0.0345	0.4528	-0.6041	0.5179	-0.1832
Libido change	0.6439	0.3513	0.6982	-0.1125	0.2278	0.0937
Poor appetite	0.3597	0.2830	0.1674	0.1735	0.2266	0.3303
Increased appetite	0.2474	0.3142	0.7407	-0.1977	0.2441	0.0765
Nervousness	0.0987	0.0244	0.8230	0.4121	-0.4224	0.0550
Constipation	0.3242	0.0553	0.0472	0.2357	-0.4673	-0.4149
Nausea	0.5528	1.0000	0.6329	0.1202	0.0000	0.1163
Tantrums	0.6439	0.1687	0.1834	-0.1125	0.3313	0.3199
Skin reactions	0.3242	0.1834	0.7440	0.2357	-0.3199	-0.0791
Hallucinations	-	-	-	-	-	-

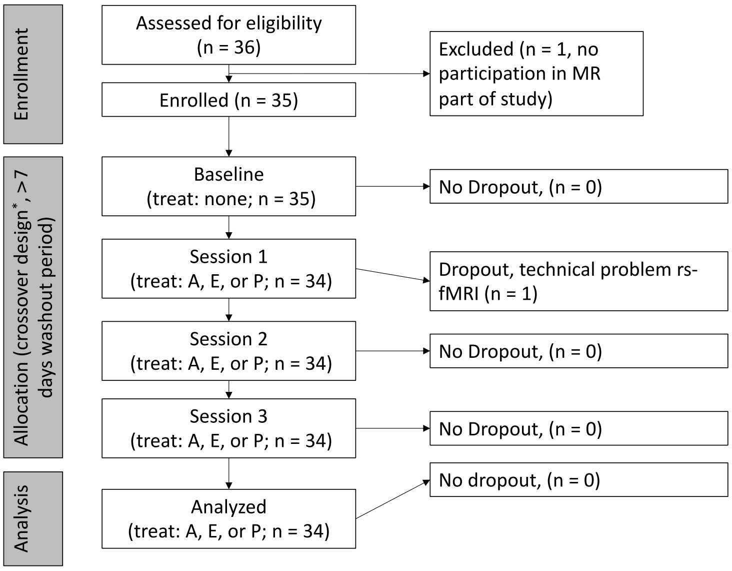

Figure S1: CONSORT flow diagram. Treatment with alprazolam (A), etifoxine (E), or placebo (P) started 5 days before a respective session. Counterbalanced order of treatments: AEP (n = 5), APE (n = 6), EAP (n = 6), EPA (n = 5), PAE (n = 6), PEA (n = 6).

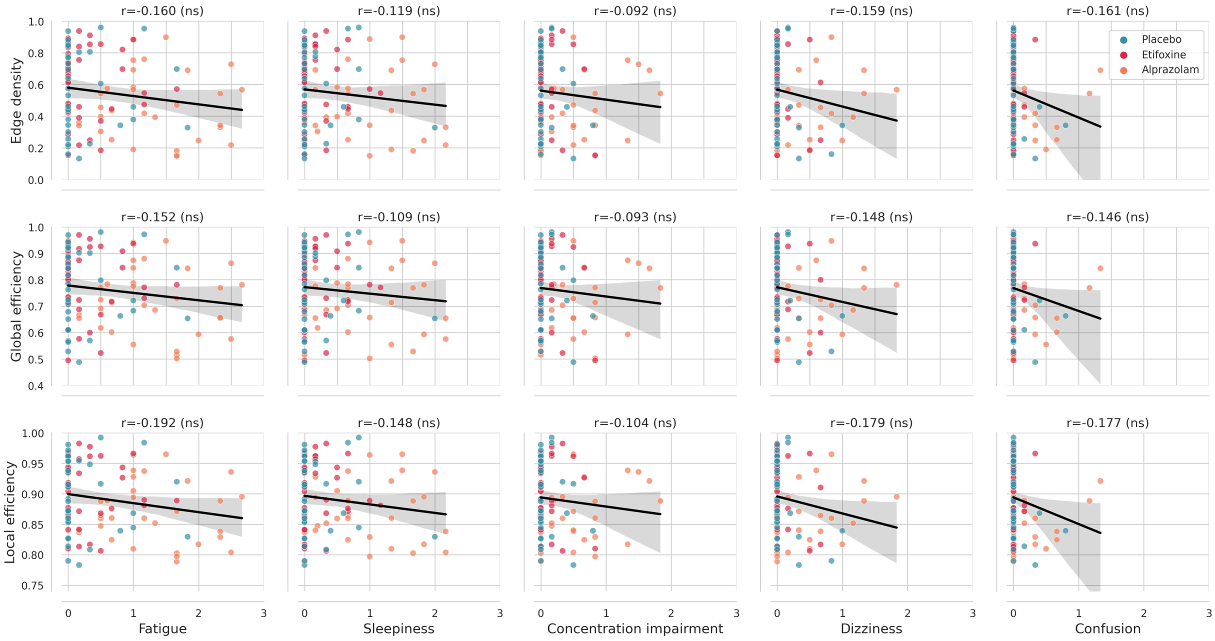

Figure S2: Relationship between functional connectivity edge density, global efficiency and local efficiency and self-reported side-effects of the participants. All graph measures were computed for a moderate thereshold of $\sigma = 0.6$ and Pearson correlation values r are shown above the respective graphs. Subjects which report stronger side-effects demonstrate a not significant (ns) but relatively consistent tendency of reduced connectivity edge-density, global efficiency and local efficiency.

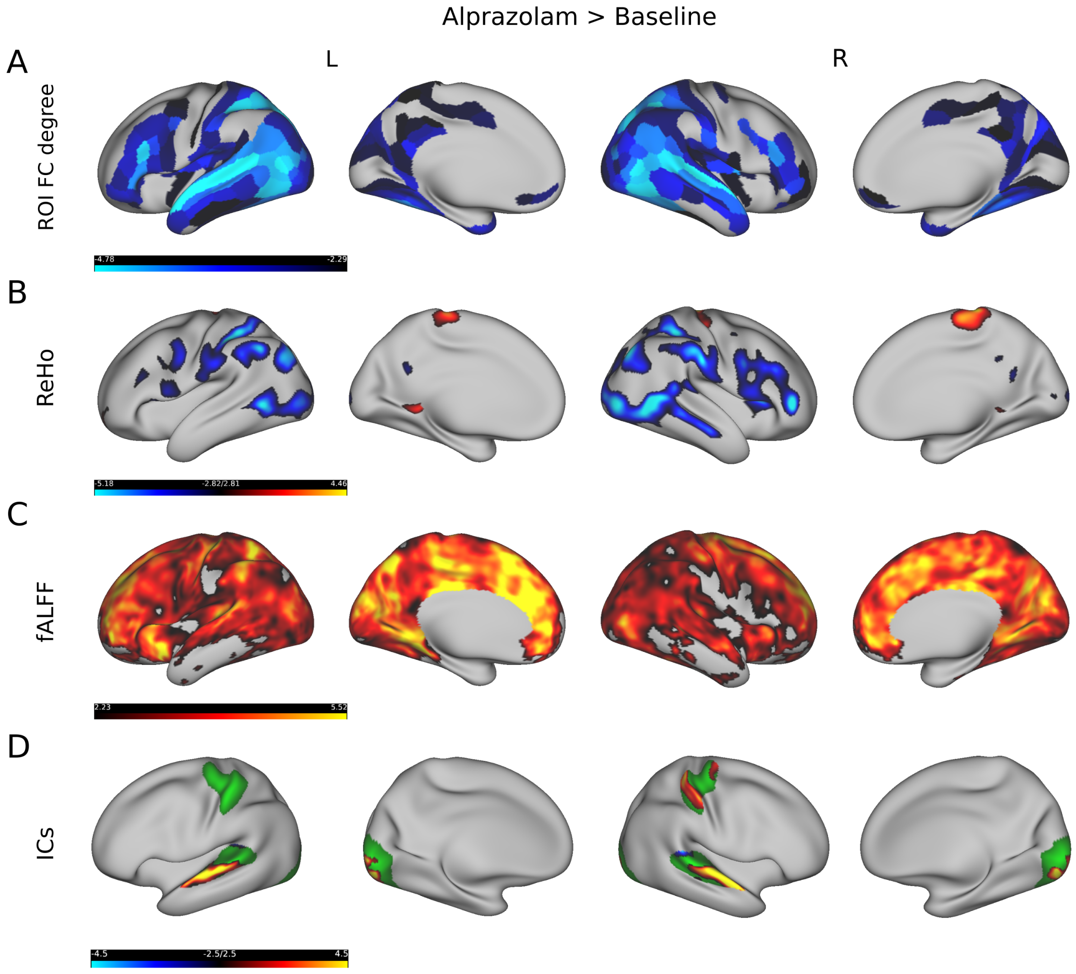

Figure S3: Comparison of rs-fMRI measures between alprazolam and baseline. The first row (A) shows that FC degree was significantly lower after administering alprazolam compared to the baseline condition. The second row (B) depicts several regions in which local connectivity, as defined by ReHo, was lower after administering alprazolam compared to baseline, except for one region in superior/medial aspects of somato-motor cortex. The third row (C) shows that low frequency amplitudes, as derived from fALFF, which were higher after administering alprazolam compared to the baseline condition. The fourth row (D) illustrate alterations in ICA based resting-state networks, which display considerable higher connectivity after the administration of alprazolam in the temporal, occipital and right somatosensory cortex. Yellow and blue depict t-values (from blue, alprazolam $<$ baseline, to yellow, alprazolam $>$ baseline).

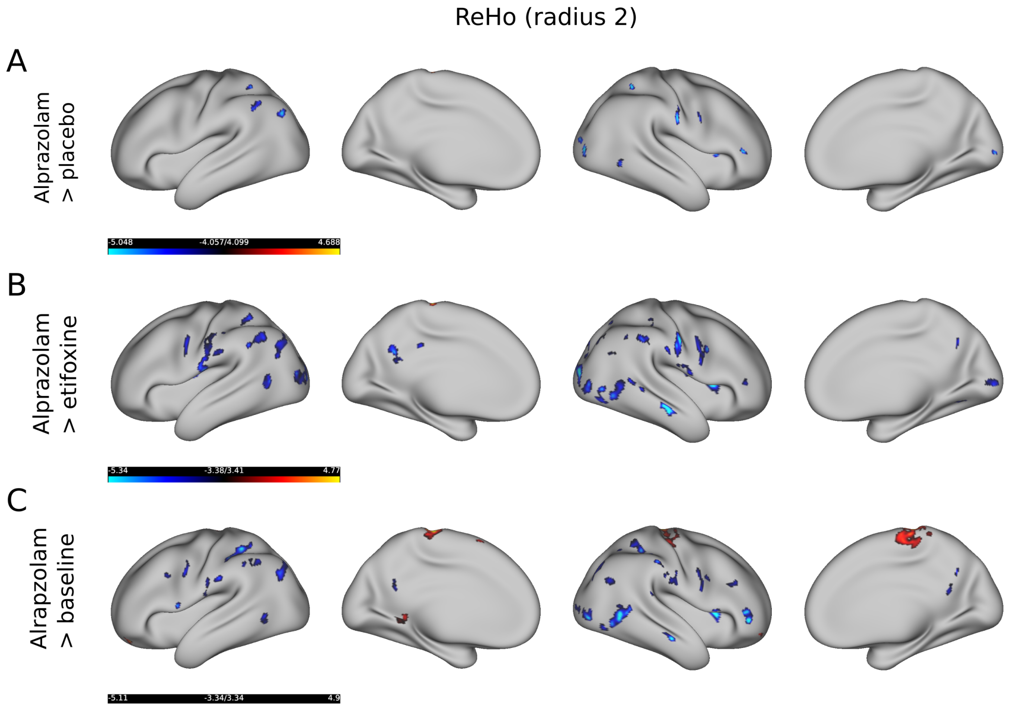

Figure S4: Comparison of ReHo of the alprazolam condition with the placebo (A), etifoxine (B) and baseline (C) condition, based on a small 2 vertices neighbourhood radius ($\approx 2.6mm$). Similar to ReHo computed with a 4 vertices radius, it was mainly lower after administering alprazolam compared to placebo, etifoxine and baseline, but increased in the superior/medial aspects of somato-motor cortex. Colors depict t-values (blue indicating alprazolam $<$ placebo/etifoxine/baseline).

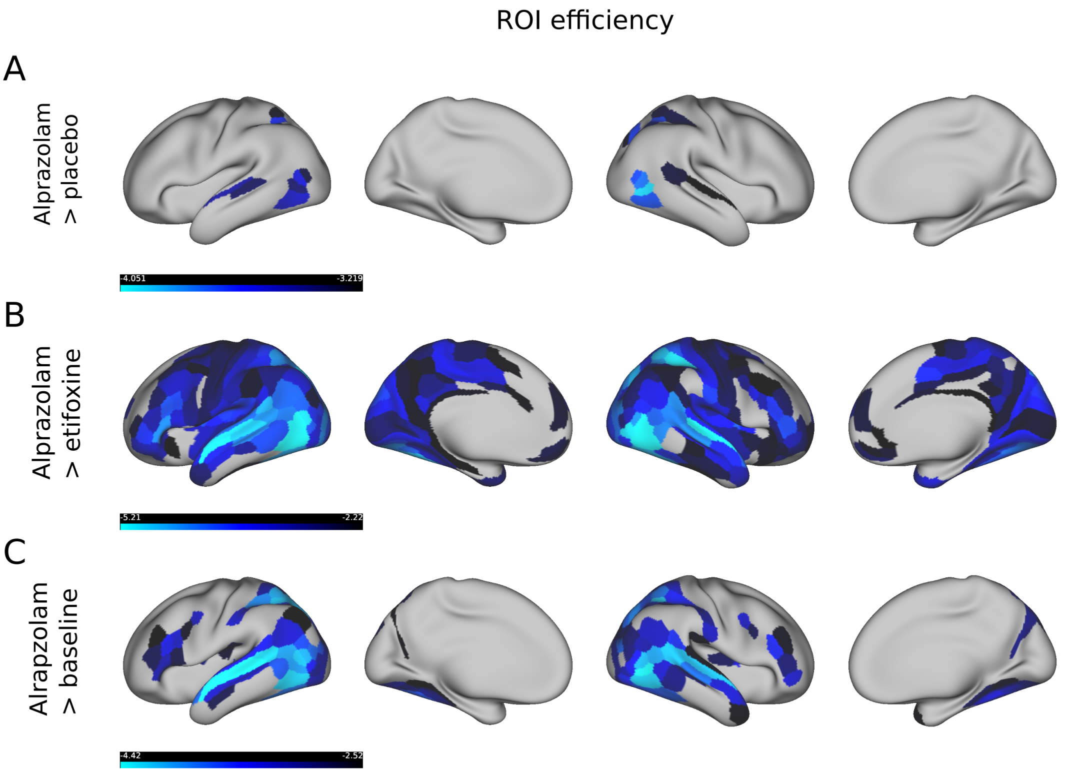

Figure S5: Comparison of ROI efficiency of the alprazolam condition with the placebo (A), etifoxine (B) and baseline (C) condition. It can observed that ROI efficiency within the functional network is significantly lowered after administration of alprazolam in comparison to all other conditions. Colors depict t-values (blue indicating alprazolam < placebo/etifoxine/baseline).

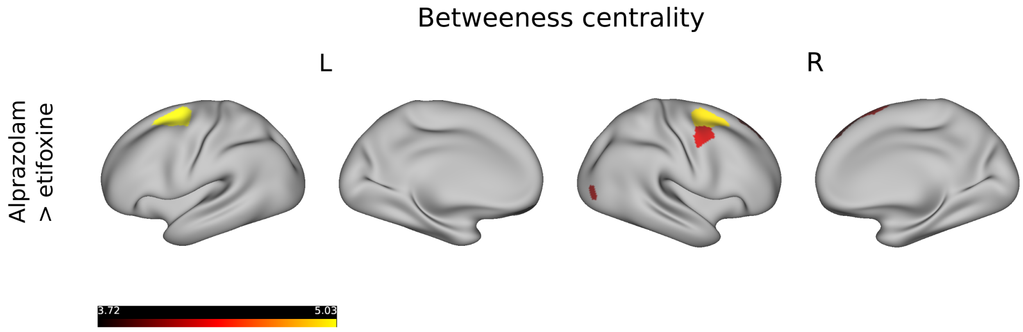

Figure S6: Regions with a significantly increased betweenness centrality in the alprazolam condition in comparison to etifoxine. Colors depict t-values (yellow indicating alprazolam > etifoxine).

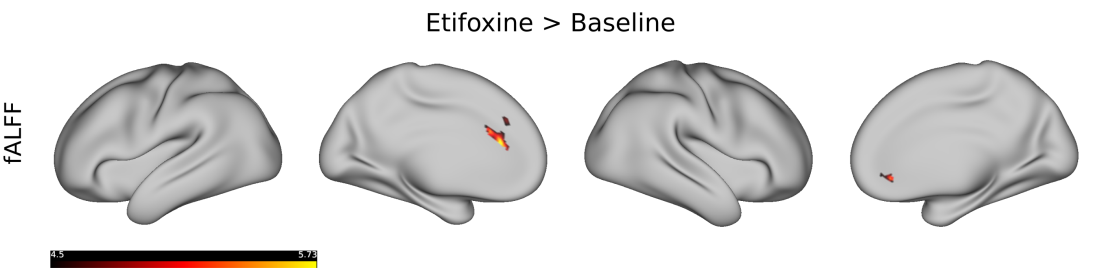

Figure S7: Comparison of fALFF values between etifoxine and baseline. Low frequency amplitudes were higher after administering etifoxine compared to the baseline condition in the anterior cingulate cortex. Yellow colors depict t-values.

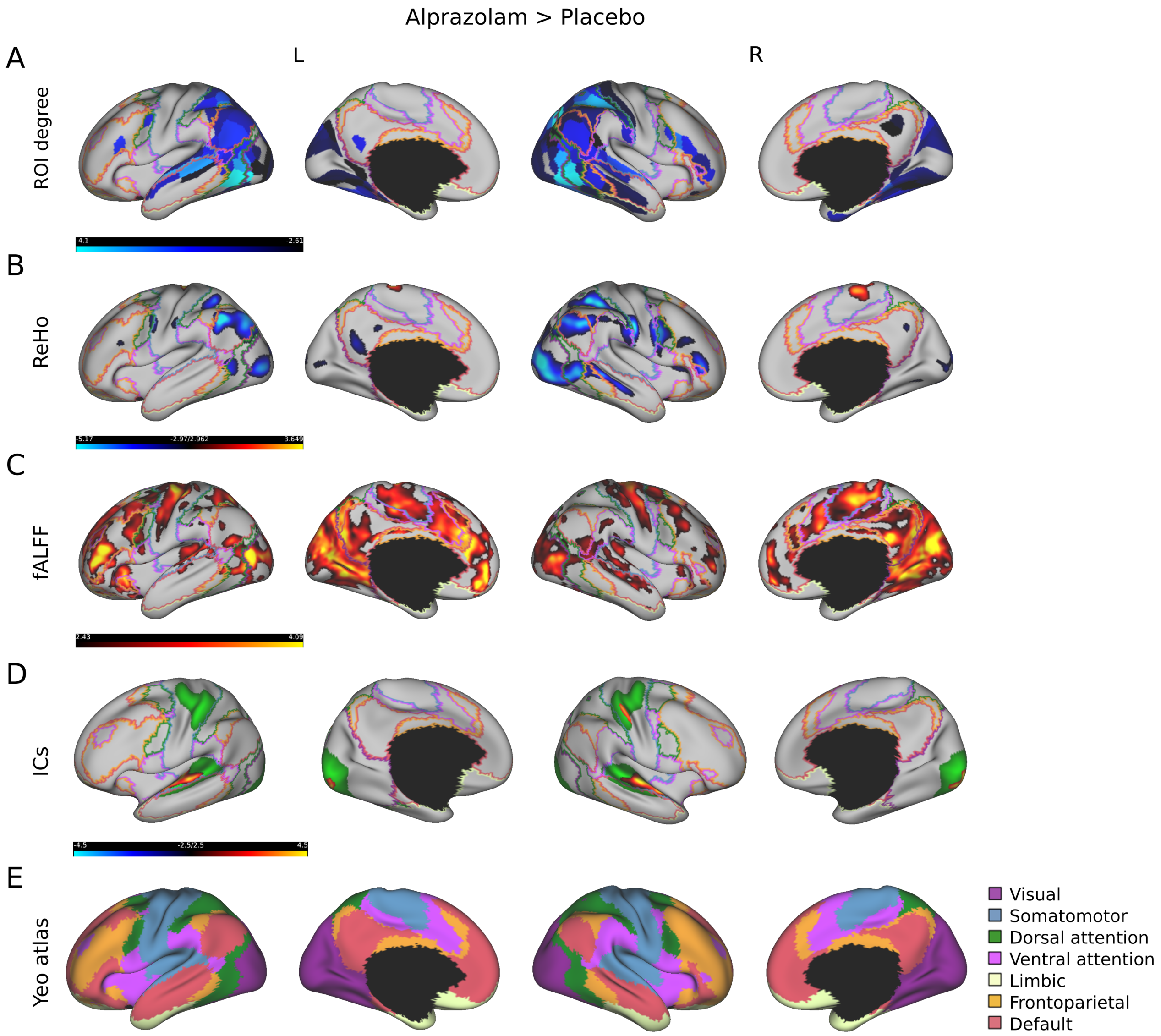

Figure S8: Comparison of rs-fMRI measures between alprazolam and placebo, including an overlay of 7 resting-state fMRI networks defined by Yeo et al. [12].

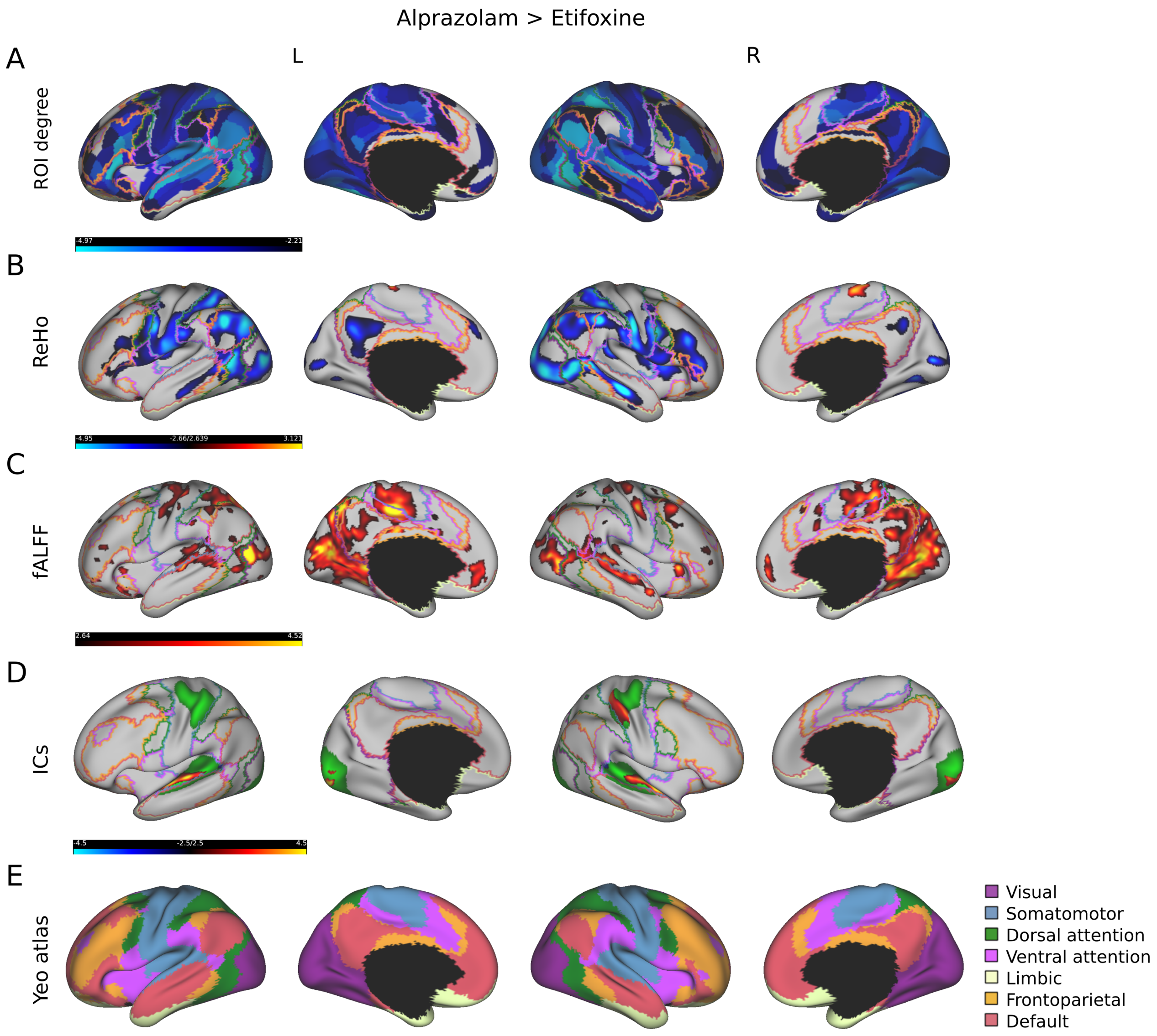

Figure S9: Comparison of rs-fMRI measures between alprazolam and etifoxine, including an overlay of 7 resting-state fMRI networks defined by Yeo et al. [12].

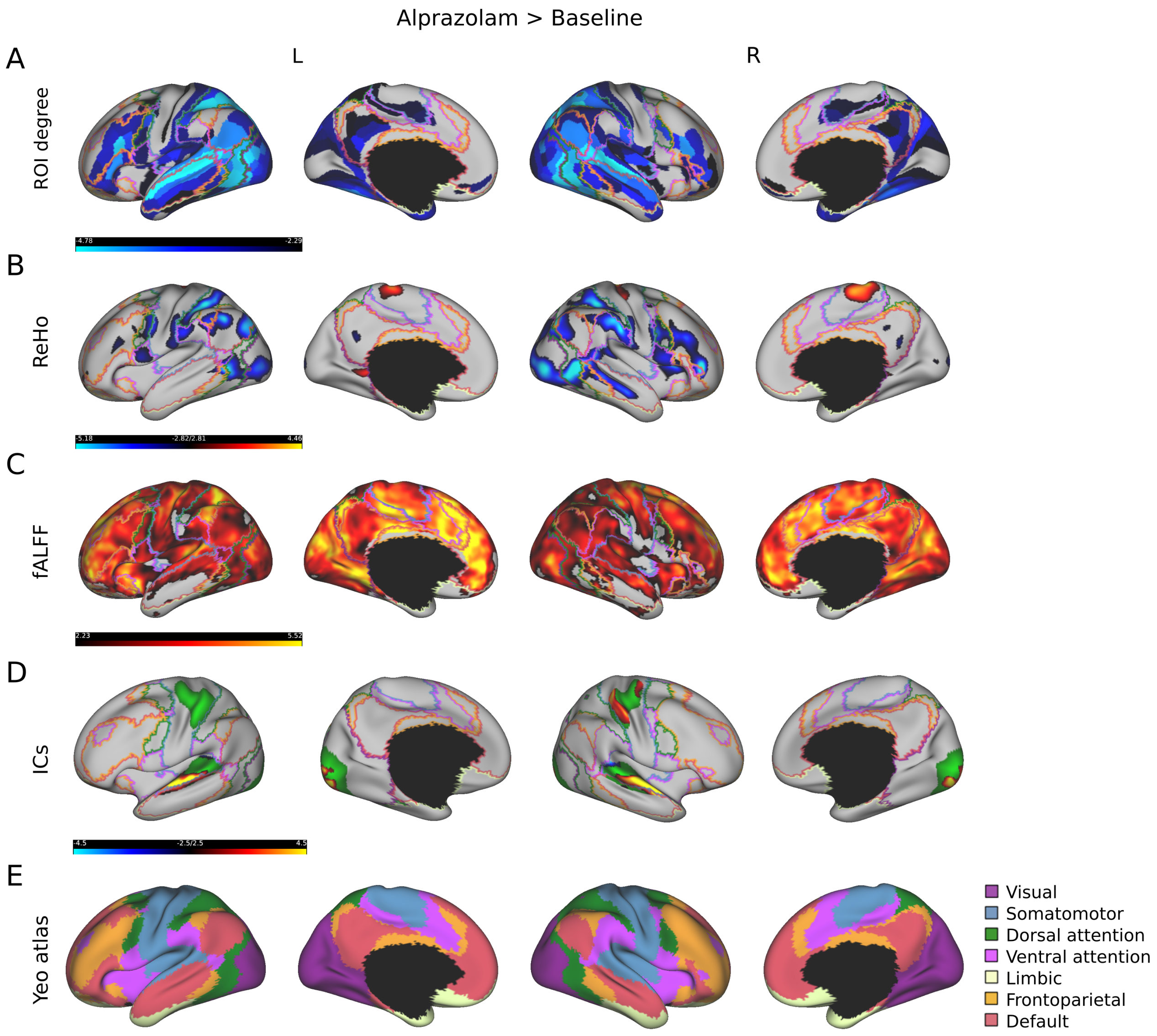

Figure S10: Comparison of rs-fMRI measures between alprazolam and baseline, including an overlay of 7 resting-state fMRI networks defined by Yeo et al. [12].

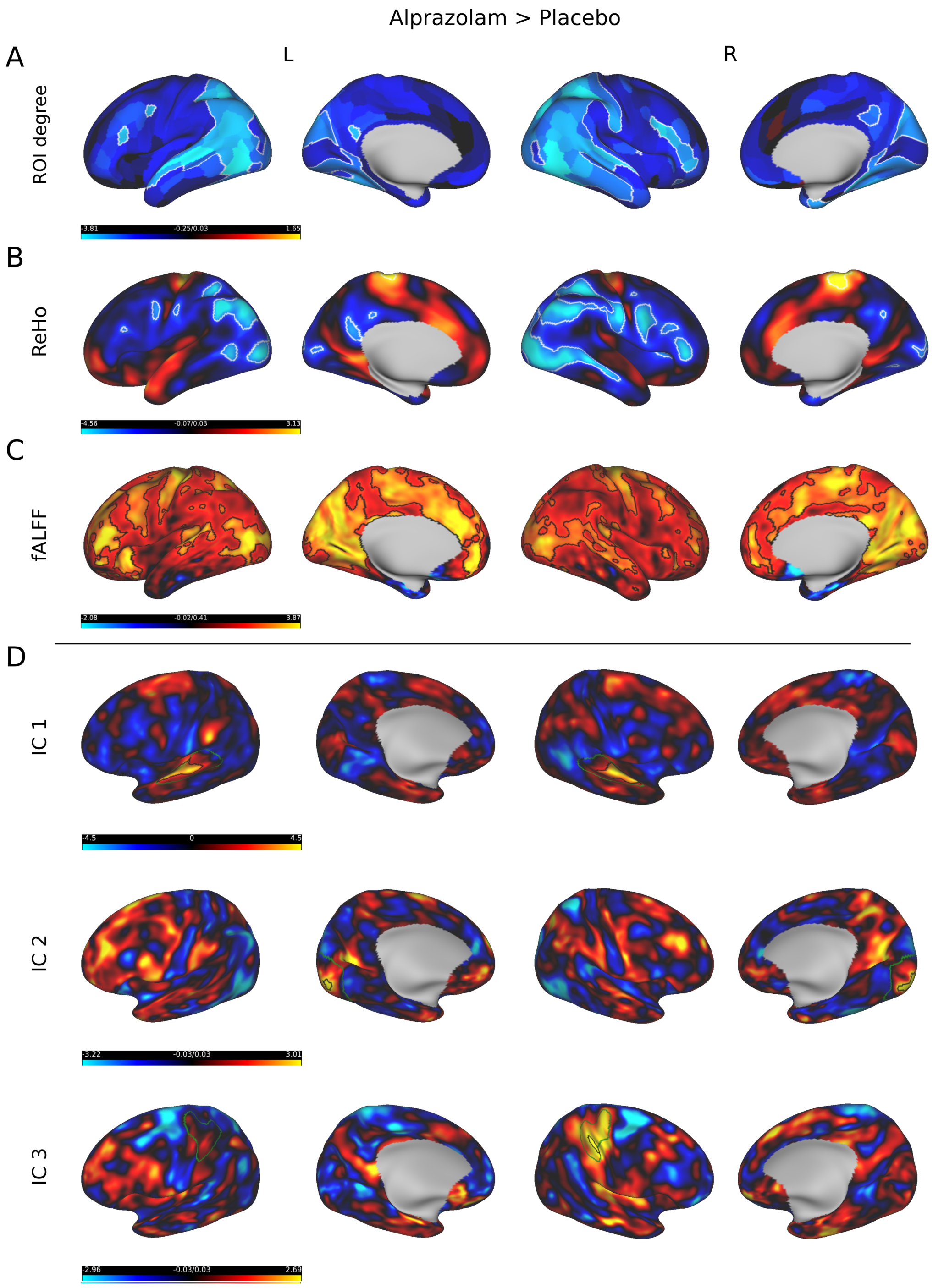

Figure S11: All t-values of rs-fMRI measures from the comparison between alprazolam and placebo. The first row (A) shows that FC degree was in general lower after administering alprazolam compared to placebo. The second row (B) shows that ReHo was mostly lower after administering alprazolam compared to placebo, but higher in superior/medial aspects of somatomotor, parieto-occipital and superior temporal cortex. The third row (C) shows that fALFF values were in general higher after administering alprazolam compared to placebo. The last three rows (D) illustrate that ICA based resting-state networks display higher activity coherence after the administration of alprazolam in the temporal, occipital and right primary somatosensory cortex. Yellow and blue depict t-values (from blue, alprazolam $<$ placebo, to yellow, alprazolam $>$ placebo). White/black outlines mark regions that significantly differ between conditions. Green outlines mark regions of average resting-state networks with $|z| > 2$.

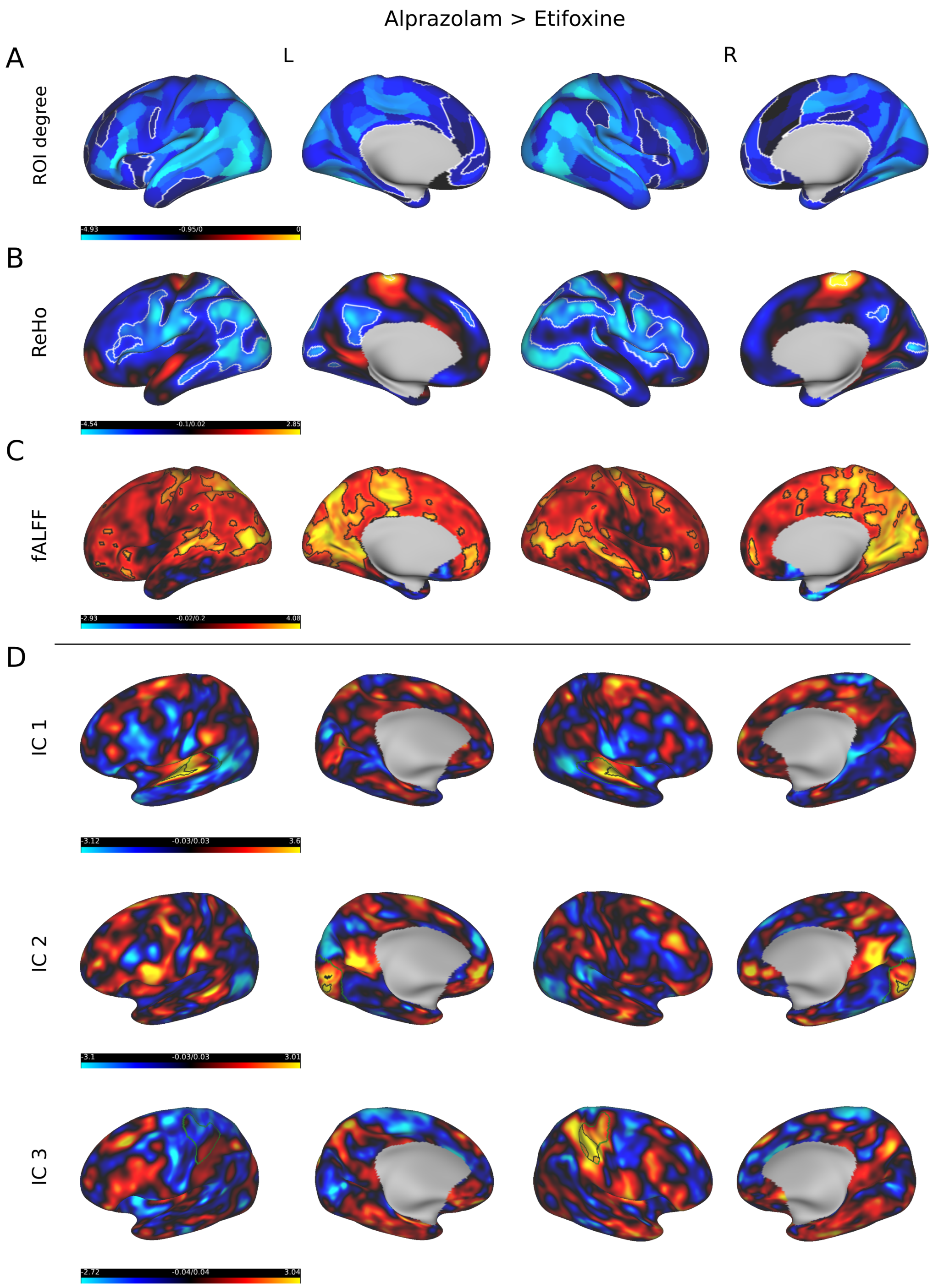

Figure S12: All t-values of rs-fMRI measures from the comparison between alprazolam and etifoxine. The first row (A) shows that FC degree was in general lower after administering alprazolam compared to the etifoxine condition. The second row (B) illustrates that ReHo was mostly lower after administering alprazolam compared to etifoxine, but higher in superior/medial aspects of somato-motor, parieto-occipital and superior temporal cortex. The third row (C) shows that fALFF values were higher after administering alprazolam compared to the etifoxine condition. The last three rows (D) illustrate that ICA based resting-state networks display higher activity coherence after the administration of alprazolam mainly in the temporal, occipital and right primary somatosensory cortex. Yellow and blue depict t-values (from blue, alprazolam $<$ etifoxine, to yellow, alprazolam $>$ etifoxine). White/black outlines mark regions that significantly differ between conditions. Green outlines mark regions of average resting-state networks with $|z| > 2$.

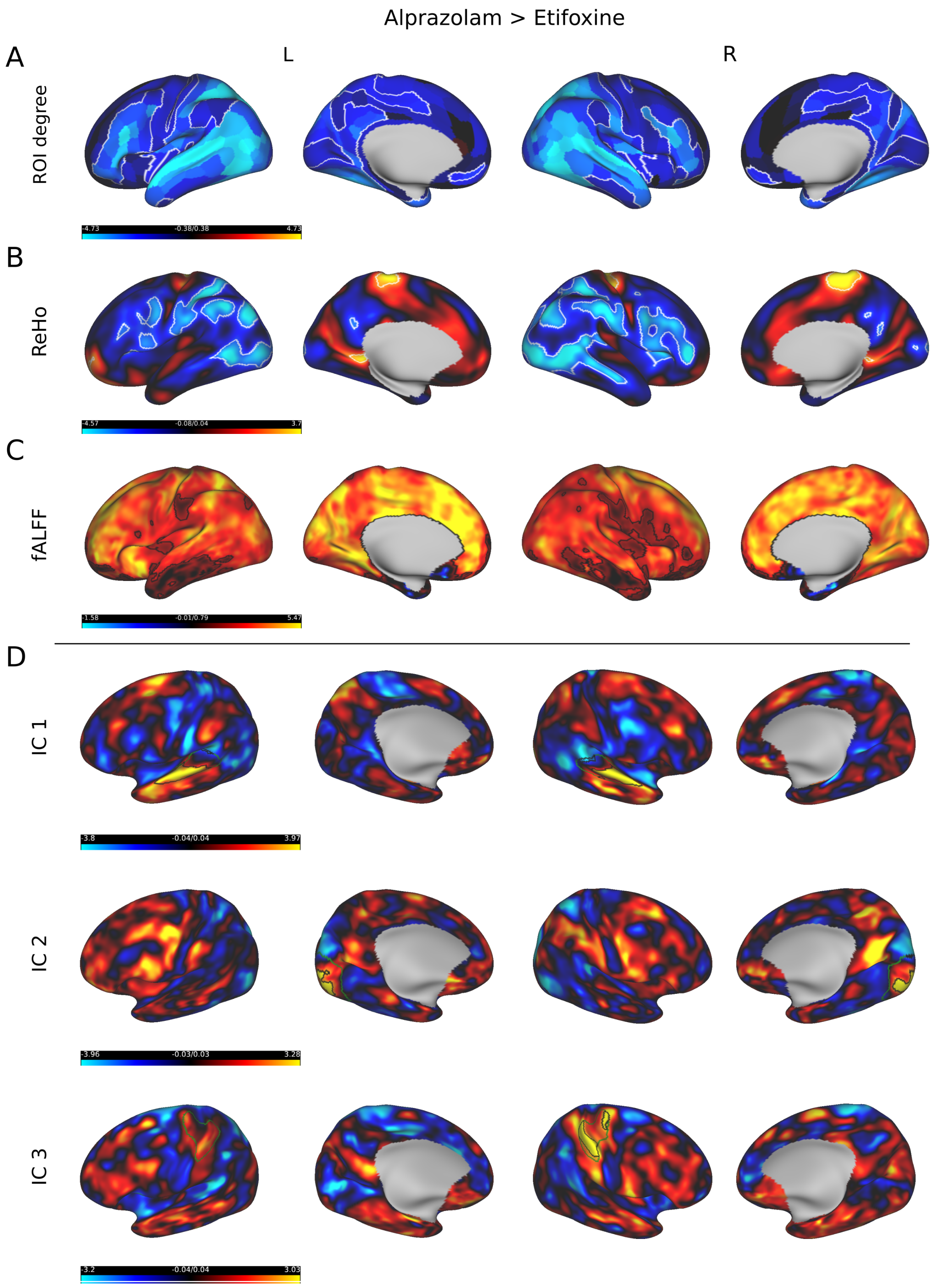

Figure S13: All t-values of rs-fMRI measures from the comparison between alprazolam and baseline. The first row (A) shows that FC degree was in general lower after administering alprazolam compared to baseline. The second row (B) shows that ReHo was mostly lower after administering alprazolam compared to baseline, but higher in superior/medial aspects of somato-motor, parieto-occipital and superior temporal cortex. The third row (C) illustrates that fALFF values were higher after administering alprazolam compared to the baseline condition. The last three rows (D) show that ICA based resting-state networks display higher activity coherence after the administration of alprazolam in the temporal, occipital and right primary somatosensory cortex. Yellow and blue depict t-values (from blue, alprazolam $<$ baseline, to yellow, alprazolam $>$ baseline). White/black outlines mark regions that significantly differ between conditions. Green outlines mark regions of average resting-state networks with $|z| > 2$.

References

[1] S. Achard and E. Bullmore. Efficiency and cost of economical brain functional networks. *PLOS Computational Biology*, 3(2):1–10, 02 2007.

[2] A. Hyvärinen. New approximations of differential entropy for independent component analysis and projection pursuit. In *NIPS*, 1997.

[3] A. Hyvärinen and E. Oja. A Fast Fixed-Point Algorithm for Independent Component Analysis. *Neural Computation*, 9(7):1483–1492, 07 1997.

[4] V. J. Kiviniemi, H. Haanpää, J.-H. Kantola, J. Jauhiainen, V. Vainionpää, S. Alahuhta, and O. Tervonen. Midazolam sedation increases fluctuation and synchrony of the resting brain BOLD signal. *Magnetic Resonance Imaging*, 23(4):531–537, 2005.

[5] J. Karhunen, E. Oja, L. Wang, R. Vigário, and J. Joutsensalo. A class of neural networks for independent component analysis. *IEEE transactions on neural networks*, 8 3:486–504, 1997.

[6] V. Kiviniemi, J. Jauhiainen, O. Tervonen, E. Pääkkö, J. Oikarinen, V. Vainionpää, H. Rantala, and B. Biswal. Slow vasomotor fluctuation in fMRI of anesthetized child brain. *Magnetic Resonance in Medicine*, 44(3):373–378, 2000.

[7] V. Kiviniemi, J.-H. Kantola, J. Jauhiainen, A. Hyvärinen, and O. Tervonen. Independent component analysis of nondeterministic fMRI signal sources. *NeuroImage*, 19(2):253–260, 2003.

[8] W. Lu and J. C. Rajapakse. ICA with reference. *Neurocomputing*, 69(16):2244–2257, 2006. Brain Inspired Cognitive Systems.

[9] J. J. McAuley, L. da Fontoura Costa, and T. S. Caetano. Rich-club phenomenon across complex network hierarchies. *Applied Physics Letters*, 91(8):084103, 08 2007.

[10] O. Sporns. *Networks of the Brain*. MIT Press, Cambridge, MA, 2010.

[11] M. Tahedl and J. V. Schwarzbach. An updated and extended atlas for corresponding brain activation during task and rest. *Human Brain Mapping*, (in press), 2023.

[12] B. T. Thomas Yeo, F. M. Krienen, J. Sepulcre, M. R. Sabuncu, D. Lashkari, M. Hollinshead, J. L. Roffman, J. W. Smoller, L. Zöllei, J. R. Polimeni, B. Fischl, H. Liu, and R. L. Buckner. The organization of the human cerebral cortex estimated by intrinsic functional connectivity. *Journal of Neurophysiology*, 106(3):1125–1165, 2011.

[13] Y.-F. Zang, T. Jiang, Y. Lu, Y. He, and L. Tian. Regional homogeneity approach to fmri data analysis. *NeuroImage*, 22:394–400, 06 2004.

[14] Q.-H. Zou, C.-Z. Zhu, Y. Yang, X.-N. Zuo, X.-Y. Long, Q.-J. Cao, Y.-F. Wang, and Y.-F. Zang. An improved approach to detection of amplitude of low-frequency fluctuation (ALFF) for resting-state fMRI: Fractional ALFF. *Journal of Neuroscience Methods*, 172(1):137–141, 2008.
